# Time progression and regional expression of brain oxidative stress induced by obstructive jaundice in rats

**DOI:** 10.1186/s42826-022-00146-z

**Published:** 2022-11-24

**Authors:** Konstantinos Lilimpakis, Aidona Tsepelaki, Electra Kalaitzopoulou, Dimitrios Zisimopoulos, Polyxeni Papadea, Marianna Skipitari, Athina Varemmenou, Apostolos Aggelis, Constantine Vagianos, Constantine Constantoyannis, Christos D. Georgiou

**Affiliations:** 1 Department of Medicine, Department of Neurosurgery, University of Patras, University Campus, GR26504 Rion, Achaia Patras, Greece; 2 Department of Medicine, Second Department of Propaedeutic Surgery, National and Kapodistrian University of Athens, 75 Mikras Asias str, 11527 Athens, Goudi Greece; 3 Department of Biology, University of Patras, University Campus, GR26504 Rion, Achaia Patras, Greece; 4 Department of Neurosurgery, St. Savvas Hospital, 171 Alexandras Avenue, 11522 Athens, Greece

**Keywords:** Oxidative stress, Obstructive jaundice, Protein oxidation, Protein carbonyls, Rhodamine B hydrazide, Lipid peroxidation

## Abstract

**Background:**

Obstructive jaundice induces oxidative changes in the brain parenchyma and plays significant role in clinical manifestations of hepatic encephalopathy. We aim to study the progression of the brain oxidative status over time and the differences of its pattern over the hemispheres, the brainstem and the cerebellum. We use an experimental model in rats and measuring the oxidative stress (OS) specific biomarkers protein malondialdehyde (PrMDA) and protein carbonyls (PrC = O).

**Results:**

Hyperbilirubinemia has been confirmed in all study groups as the result of common bile duct obstruction. We confirmed increase in both PrMDA and PrC = O biomarkers levels with different type of changes over time. We also confirmed that the oxidative process develops differently in each of the brain areas in study.

**Conclusions:**

The present study confirms the progressive increase in OS in all brain areas studied using markers indicative of cumulative protein modification.

**Supplementary Information:**

The online version contains supplementary material available at 10.1186/s42826-022-00146-z.

## Background

Cerebral oxidative stress (OS) is the result of the imbalance generated by the increased production of Reactive Oxygen Species (ROS) and the inability of the physiological antioxidant mechanisms to respond, leading to a cascade of further molecular reactions [1]. These changes promote cellular damage not only with the oxidation of protein and lipid structures but also directly targeting the DNA and promoting chromosomal translocation, deletions and cell cycle arrest leading to cell necrosis [2]. Various conditions have been associated with the induction of OS in the brain, including cerebrovascular disease and cerebral ischaemia, subarachnoid haemorrhage, traumatic brain injury, neurodegenerative disease such as Alzheimer, Parkinson and Huntington diseases and neuropsychiatric disorders [3]. Brain OS has also been associated with increased bilirubinemia. Previous studies have effectively shown the relation of brain tissue OS and obstructive jaundice [4]. Furthermore, there is multi-organ, time related increase of this condition proven by superoxide radical measurements. Such accumulation of this direct factor of oxidative stress was confirmed in the liver, intestine, kidneys and the brain with increasing levels over time [5]. The aim of the current study is to show the progression of OS, expressed by two OS-specific and cumulative protein oxidative damage indicators protein malondialdehyde (PrMDA) and protein carbonyls (PrC = O), in relation to the duration of the elevated bilirubin levels up to 14 days post icterus induction, and the differences of the oxidative process in different brain areas.

## Results

### Biochemical parameter and endotoxin levels

#### Comparisons among control, sham and experimental animal subgroups in all animal groups

Most of tested biochemical parameters showed similar levels between controls and sham operated animals with normal or slightly increased levels, but higher levels in animals with bile duct ligation, in all groups of the study. In particular, total bilirubin, direct bilirubin, SGOT, SGPT, GGT and ALP followed the above trend, whereas LDH, amylase and CRP did not, except an increase of CRP levels in sham operated animals and animals with bile duct ligation at 7 days after operation (Tables 1, 2, 3 and 4).

**Table 1:** Biochemical parameters among animal groups at 12-h post ligation

Βiochemical parameters (mean ± SD)	Control	Sham	Ligation	*p*
Total bilirubin (mg/dl)	0.12 ± 0.01	0.19 ± 0.03	3.56 ± 0.66	< 0.001
Direct bilirubin (mg/dl)	0.02 ± 0	0.085 ± 0.035	3.33 ± 0.59	< 0.001
SGOT (IU/L)	104 ± 17	150.5 ± 32.5	2007.9 ± 1393.1	0.005
SGPT (IU/L)	32.5 ± 2.5	41.5 ± 2.5	1202.8 ± 363.9	0.001
GGT (IU/L)	1.5 ± 0.5	4.5 ± 3.5	11 ± 4	0.03
ALP (IU/L)	153.5 ± 0.5	112.5 ± 12.5	642.9 ± 193.4	0.005
LDH (IU/L)	1123 ± 515	2093 ± 624	1970.4 ± 848.7	0.47
Amylase (IU/L)	2309.5 ± 183.5	1869 ± 88	1494.6 ± 221.9	0.004
CRP (mg/dl)	0.1 ± 0	0 ± 0	0 ± 0	0.004

**Table 2:** Biochemical parameters among animal groups at 2-day after post ligation

Βiochemical parameters	Sham	Ligation	*p*
Total bilirubin (mg/dl) [median (min–max)]	0.14 (0.13–0.14)	8.9 (7.87–17.42)	0.025
Direct bilirubin (mg/dl) (mean ± SD)	0.04 ± 0.01	9.6 ± 2.66	0.002
SGOT (IU/L) (mean ± SD)	130 ± 21	995 ± 299.2	0.005
SGPT (IU/L) (mean ± SD)	39.5 ± 4.5	843.7 ± 311.3	0.01
GGT (IU/L) (mean ± SD)	1 ± 0	15 ± 3.1	0.001
ALP (IU/L) (mean ± SD)	117.5 ± 5.5	609.8 ± 169.4	0.007
LDH (IU/L) (mean ± SD)	2111 ± 882	1359.7 ± 662	0.47
Amylase (IU/L) (mean ± SD)	1785.5 ± 189.5	1870.2 ± 330	0.278
CRP (mg/dl) (mean ± SD)	0.06 ± 0.06	0 ± 0	0.014

**Table 3:** Biochemical parameters among animal groups at 7-day post ligation

Βiochemical parameters	Sham	Ligation	*p*
Total bilirubin (mg/dl) (mean ± SD)	0.1 ± 0	13.86 ± 2.02	< 0.001
Direct bilirubin (mg/dl) (mean ± SD)	0.01 ± 0	11.61 ± 1.63	< 0.001
SGOT (IU/L) [median (min–max)]	141.5 (141–142)	490 (253–1275)	0.019
SGPT (IU/L) [median (min–max)]	49.5 (48–51)	127 (91–361)	0.019
GGT (IU/L) (mean ± SD)	2 ± 0	36.7 ± 11.4	0.001
ALP (IU/L) (mean ± SD)	122 ± 2	475.6 ± 52.9	< 0.001
LDH (IU/L) (mean ± SD)	2273.5 ± 29.5	1856.6 ± 871.8	0.421
Amylase (IU/L) (mean ± SD)	2084.5 ± 35.5	1875.6 ± 192.3	0.048
CRP (mg/dl) (mean ± SD)	6.05 ± 0.45	5.3 ± 2	0.013

**Table 4:** Biochemical parameters among animal groups at 14-day post ligation

Βiochemical parameters	Sham	Ligation	*p*
Total bilirubin (mg/dl) (mean ± SD)	0.08 ± 0.03	11.77 ± 2.33	< 0.001
Direct bilirubin (mg/dl) (mean ± SD)	0.09 ± 0.01	9.32 ± 1.81	< 0.001
SGOT (IU/L) (mean ± SD)	129.5 ± 8.5	459.9 ± 56.2	< 0.001
SGPT (IU/L) (mean ± SD)	63 ± 6	112 ± 16.5	< 0.001
GGT (IU/L) (mean ± SD)	3 ± 2	60.3 ± 15.6	< 0.001
ALP (IU/L) (mean ± SD)	150.5 ± 14.5	479.8 ± 100.9	< 0.001
LDH (IU/L) (mean ± SD)	1281 ± 435	1641 ± 608.2	0.538
Amylase (IU/L) (mean ± SD)	1897.5 ± 420.5	1858.8 ± 159.8	0.125
CRP (mg/dl) [median (min–max)]	0 (0–0)	0 (0–0)	0.002

#### Comparisons among animals with bile duct ligation at various time points

Different patterns of level variations were noted as time was passing among the tested biochemical parameters in animals with bile duct ligation. Total bilirubin, direct bilirubin and CRP reached their peak at 7 days after operation (Table 5), whereas SGOT, SGPT and ALP at 12 h after operation. The highest levels of GGT were detected at 14 days after operation. On the other hand, amylase levels remained relatively stable after an increase found at 2-day after ligation of bile duct, while LDH levels did not show significant variation.

**Table 5:** Bilirubin levels comparison among animals with bile duct ligation at various time points

Βiochemical parameters	12-h	2-day	7-day	14-day	*p*
Total bilirubin (mg/dl) [median (min–max)]	3.48 (2.81–4.81)	8.9 (7.87–17.42)	12.86 (11.43–17.25)	11.57 (8.2–16)	< 0.001
Direct bilirubin (mg/dl) (mean ± SD)	3.33 ± 0.59	9.6 ± 2.66	11.61 ± 1.63	9.32 ± 1.81	< 0.001
CRP (mg/dl) (mean ± SD)	0 ± 0	0 ± 0	5.3 ± 2	0 ± 0	p < 0.001

#### Comparisons among animals with bile duct ligation in terms of endotoxin levels

No statistically significant variations were found during post ligation time, regarding endotoxin levels in peripheral blood, blood from aorta or blood from portal vein among animals with bile duct ligation, although the peak levels for all were noted at 7 days after operation. Furthermore, no statistically significant variations of endotoxin levels in blood samples from periphery, aorta and portal vein were observed at each time point (Table 6).

**Table 6:** Comparisons among animals with bile duct ligation in endotoxin levels

Peripheral blood [median (min–max)], *p* = 0.755			
12-h: 6.9 (5.7–20.8)	2-day: 7.3 (5.5–17.1)	7-day: 8.3 (5.5–25.9)	14-day: 5.9 (5.1–24.9)
Aorta (mean ± SD), *p* = 0.102			
12-h: 6.61 ± 1.16	2-day: 7.03 ± 1.26	7-day: 8.83 ± 4.25	14-day: 5.76 ± 0.51
Portal vein (mean ± SD), *p* = 0.071			
12-h: 6.76 ± 1.09	2-day: 7.72 ± 1.85	7-day: 11.8 ± 8.56	14-day: 5.79 ± 0.78
12-h [median (min–max)], *p* = 0.246			
peripheral: 6.9 (5.7–20.8)	Aorta: 6.2 (5.5–8.6)	Portal: 6.4 (5.6–8.8)	
2-day [median (min–max)], *p* = 0.836			
peripheral: 7.3 (5.5–17.1)	Aorta: 7.2 (5.2–9.1)	Portal: 7.2 (5.2–10.7)	
7-day [median (min–max)], *p* = 0.834			
peripheral: 8.3 (5.5–25.9)	Aorta: 8.25 (5–17.8)	Portal: 7.6 (4.8–30.9)	
14-day [median (min–max)], *p* = 0.571			
peripheral: 5.9 (5.1–24.9)	Aorta: 5.7 (5.2–6.7)	Portal: 5.7 (4.9–7.7)	

### OS evaluation

**Fig. 1:**
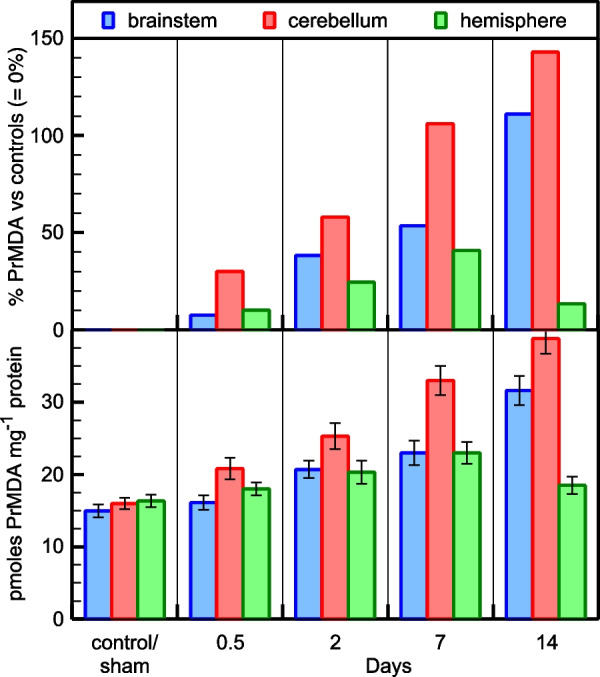
Oxidative stress evaluation in the rat brainstem, cerebellum, hemisphere among animals with bile duct ligation at various time points (versus control and sham). Protein oxidative modification after protein (Pr) reaction with the end product of peroxidized lipids MDA. Data are expressed as mean ± SD PrMDA pmoles per mg total protein (bottom), and as % in comparison to control/sham set at 0% (top)

**Fig. 2:**
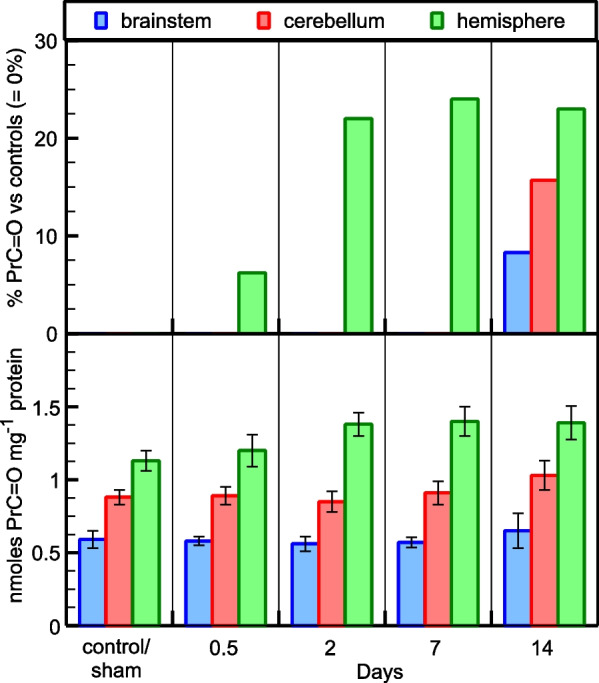
Oxidative stress evaluation in the rat brainstem, cerebellum, hemisphere among animals with bile duct ligation at various time points (versus control and sham). Free radical-induced protein oxidative modification in the form of protein carbonyls (PrC = O). Data are expressed as mean ± SD PrMDA nmoles per mg total protein (bottom), and as % in comparison to control/sham set at 0% (top)

It is shown that bile duct ligation causes OS in the rat brainstem, cerebellum, and hemisphere among animals with bile duct ligation at various time points, exhibited at least at 14-day post ligation (Tables 7 and 8; control and sham are presented as one group since they have statistically the same PrMDA and PrC = O levels). PrMDA levels show a gradual increase over time in all three brain areas tested, with more pronounced (above 100%) in brainstem and cerebellum (Fig. 1). In terms of statistical analysis, the *ANOVA* test revealed that hemisphere PrMDA levels are statistically increased [F(4, 10) = 13.18, *p* = 0.01, η_p_^2^ = 0.84] two days after bile duct ligation (compared to control/sham; mean difference = −2.73, *p* = 0.01). Furthermore, the *ANOVA* test revealed that brainstem PrMDA levels are significantly increased [F(4, 10) = 67.50, *p* < 0.01, η_p_^2^ = 0.96] two days after bile duct ligation (compared to control/sham; mean difference = −6.03, *p* < 0.01), and also between 7 and 14 days after bile duct ligation (mean difference = −8.53, *p* < 0.01). Additionally, the *ANOVA* test revealed that cerebellum PrMDA levels are significantly increased [F(4, 10) = 84.29, *p* < 0.01, η_p_^2^ = 0.97] two days after bile duct ligation (compared to control/sham; mean difference = −9.97, *p* < 0.01), and also between 2 and 7 days after bile duct ligation (mean difference = −7.80, *p* < 0.01). Moreover, the *ANOVA* test for the PrC = O levels revealed that they are significantly increased [F(4,10) = 4.04, *p* = 0.033, η_p_^2^ = 0.62] only in rats’ hemisphere two days after bile duct ligation (compared to control/sham; mean difference = −0.25, *p* = 0.04). (Fig. 2). In rats’ brainstem and cerebellum, in contrast, a slight increase in PrC = O levels is observed 14 days after bile duct ligation, which however is not statistically important in both cases (Fig. 2). It should be noted that PrC = O levels (Fig. 2) reach a plateau (20% over control/sham) in the hemisphere starting at 2-day, while in brainstem and cerebellum the increase (~ 9% and ~ 16%, respectively) appears only at 14-d.

**Table 7:** PrMDA levels

	M ± SD
*Hemisphere*	
Control/Sham	16.03 (± 0.84)
12 h	17.6 (± 0.78)
2 days	20.97 (± 2.08)
7 days	23.4 (± 1.51)
14 days	18.77 (± 1.25)
*Brainstem*	
Control/Sham	15 (± 1.11)
12 h	16.2 (± 1.15)
2 days	21.03 (± 1.36)
7 days	23.67 (± 1.53)
14 days	32.2 (± 1.95)
*Cerebellum*	
Control/Sham	16 (± 1.00)
12 h	20.97 (± 1.74)
2 days	25.97 (± 2.00)
7 days	33.77 (± 2.17)
14 days	38.80 (± 1.59)

PrMDA levels in the rat brainstem, cerebellum and hemisphere among animals with bile duct ligation at various time points (versus control and sham). Data are expressed as mean ± SD PrMDA pmoles per mg total protein

Since control and sham present the same PrMDA levels, we present them as one group

**Table 8:** PrC = O levels

	M ± SD
*Hemisphere*	
Control/Sham	1.13 (± 0.56)
12 h	1.18 (± 0.13)
2 days	1.38 (± 0.87)
7 days	1.40 (± 0.10)
14 days	1.38 (± 0.15)
*Brainstem*	
Control/Sham	0.59 (± 0.07)
12 h	0.57 (± 0.02)
2 days	0.57 (± 0.07)
7 days	0.56 (± 0.04)
14 days	0.64 (± 0.13)
*Cerebellum*	
Control/Sham	0.88 (± 0.07)
12 h	0.89 (± 0.08)
2 days	0.86 (± 0.09)
7 days	0.90 (± 0.087)
14 days	1.06 (± 0.11)

PrC = O levels in the rat brainstem, cerebellum and hemisphere among animals with bile duct ligation at various time points (versus control and sham). Data are expressed as mean ± SD PrC = O nmoles per mg total protein

Since control and sham present the same PrMDA levels, we present them as one group

## Discussion

The disruption of blood brain barrier (BBB) is considered fundamental for the clinical manifestations of hepatic encephalopathy. The associated mechanisms include matrix metalloproteinase-9 (MMP-9) and tissue inhibitor of matrix metalloproteinases (TIMPs) activation and simultaneous loss of tight junction proteins such as occluding, claudin-5 and ZO-1 [6, 7]. There is evidence that hypoxia induced oxidative stress alters the BBB permeability with alteration in localization and structure of occludin [8]. Similarly, the occludin downregulation under OS post obstructive jaundice affects the brain capillaries by disrupting the BBB and it is also time dependent, directly associated with the increase of the superoxide radical level [9].

In the oxidative environment and under the effect of the increased ROS all cellular macromolecules can be affected including proteins, lipids, and nucleic acids. The lipid peroxidation MDA product is widely used as oxidative status marker, although its value as accurate biomarker has been questioned [10]. Proteins modification under such conditions to transient, reversible or irreversible products has a quite significant role considering their large quantities [11]. Protein carbonylation, although difficult, leads to irreversible protein transformation products, thus more stable and of increased value as oxidative status markers. This procedure can be further reinforced with the addition of the formed malondialdehydes [11]. There are also studies with electron microscopic evaluation showing structural changes of neurons and glial cells with neuronal apoptosis and astrocytic oedema and blood brain barrier basal membrane disruption [12]. These changes have been proven to be time related and were found irreversible after 14 days tissue exposure to the bilirubinemia induced oxidative environment [12].

In our study the levels of PrMDA show a gradual increase over time, suggesting the onset of oxidative environment as soon as 12 h post common bile duct ligation and exceeding 100% increase at 14 days post ligation in cerebellar and brainstem tissue when compared to the control/sham group. The hemisphere tissue PrMDA levels are also increased, with their levels however reaching a maximum of 40% at 7 days post ligation and slightly dropping for the group that was sacrificed at 14 days post op. The elevation of bilirubin levels corresponds to the oxidative changes, reaching a maximum at 7 days post-ligation and slightly reducing at 14 days. Similarly, PrC = O levels, as indicator of proteinaceous oxidative insult, confirm the oxidative process in all the three separate brain areas, however with milder elevation. It is also evident that the latter procedure is more notable in the hemisphere while cerebellum and brainstem only show late alterations at the 14-day post ligation group. This profile level difference between the OS markers PrMDA and PrC = O, may be attributed to the fact that MDA is produced by phospholipids that in a time fashion can peroxidize each other, as being in proximity in membrane structures especially in lipid-rich biological tissues such as brain, while protein carbonylation is considered as a slower phenomenon [13].The high sensitivity of the RBH fluorometric assay which was used for the PrC = O quantification, allowing detection limit as low as 0.4 pmol [14], confirms the start of the proteins oxidation even at 12 h post CBD ligation when compared to the control/sham group.

However, in comparison to previous studies where superoxide radical as OS marker failed to show increase in rat cerebellum of similar study models [15], the increased oxidative response in the present study, based on the PrMDA and PrC = O levels as markers, is shown in all brain areas. More specifically the cerebellar structures show significant oxidative response proven by the increased level of PrMDA. These findings reflect changes 12 h post ligation to a degree of 30% over control and sham group animals and progressively increasing over time reaching 60%, 100% and 160% over 48 h, 7 days and 14 days post ligation respectively. Of note, the superoxide radical levels of the cerebellum in sham group of the aforementioned study were significantly increased compared to the other brain areas. The discrepancy in the findings could be also explained by different ROS participation in the oxidative results and by regional differences in microcirculation or intrinsic factors that regulate certain intraneural pathways [15].

Previous studies have shown alterations of the oxidative effect in different brain areas, with the cerebellum and the hippocampus being mostly affected and the cortex tissue showing lower levels [16–18]. Alterations in antioxidants levels locally, such as glutathione and catalase [18] can support these differences.

The comparison of the chronic modification of the PrMDA and PrC = O levels is confirmative of the protein carbonylation being a slower procedure and affecting more the hemispheres. Taking also into consideration that the PrMDA formation in the hemispheres is relatively kept at lower levels, this finding could be the result of a different pace of protein degradation processes between the different brain areas, or alternatively of the differences in availability and expression of the antioxidant systems [4]. Selective vulnerability differences of neurons in various brain areas have also been proposed as a multifactorial result including local demands of ROS/RNS systems for signalling, mitochondrial dysfunction, calcium dysregulation and glutamate hyperactivity [19]. It is also interesting that the PrMDA levels in the hemisphere samples of the 14 days group stop showing an increasing pattern; instead, there is a 20% decrease when compared to the 7 days group. Apart from the possibility of localized antioxidant response, another hypothesis could be that these products may participate further in the carbonylation process in the hemispheres and support the PrC = O level changes.

## Conclusions

In conclusion, the above study shows direct time progression of the obstructive jaundice induced brain oxidative status with the measurement of reliable OS protein carbonyl products and it also shows distinction of the phenomenon progression in different brain areas. The acquired data can contribute to further research focused on the efficacy of antioxidative mechanisms or specific BBB-related protein changes with time and it can enhance the understanding of the oxidative processes in the different brain areas.

## Methods

### Animals

Fifty-two male albino Wistar rats weighting between 250–350 gr were used in this study. They were housed in stainless-steel cages, 2 rats per cage, under controlled temperature and humidity conditions with 12 h dark/light cycles and maintained on standard laboratory diet and water throughout the experiment, except for an overnight fast before the surgery. The age of the rats at the time of the experiment was between fourteen to sixteen weeks.

### Experimental design

The rats were randomly divided in 4 groups depending on the time they were sacrificed, and samples were taken post bile duct ligation: The 1st in 12 h (12-h), the 2nd in 2 days (2-day), the 3rd in 7 days (7-day) and the 4th in 14 days (14-day). Each of these groups was divided in Control, Sham and Experimental subgroups. Control and Sham subgroups numbered two animals in each group and Experimental subgroup numbered nine animals in each group. The experiment was performed according to European and National Legislation Rules for animal experiments and it obtained the license number EL25BIO05.

### Surgical procedures

The Control subgroup animals in all groups did not undergo any surgical procedure before the sacrifice and the removal of the samples. The Sham subgroup animals in all groups underwent laparotomy with bile duct mobilization and preparation without ligation. The Experiment subgroup animals in all groups underwent laparotomy with bile duct ligation and transection. All surgical procedures were performed under sterile conditions using light anesthesia with ether. The skin was prepared with 10% povidone-iodine and shaving at the incision site. Standard midline longitudinal abdominal incision was used. The common bile duct was then identified, mobilized, prepared and doubly ligated using vicryl 3–0 sutures and then divided. The abdominal wall was then closed in a two-layer fashion using vicryl 3–0 and nylon 2–0 sutures. All surgical procedures were uneventful and after short recovery time the animals were returned to the preoperative housing conditions. For each group, depending on the predefined experiment time after the jaundice induction, all the animals were operated with laparotomy, using the same conditions of anesthesia and preparation. Initially after anesthesia peripheral blood sample was obtained and then after the laparotomy further blood samples were obtained from the portal vein and the aorta. The perioperative abdominal inspection did not reveal any major incidents or concerns. The animals were sacrificed by decapitation and the brain was quickly extracted and divided into hemisphere, brainstem and cerebellum samples. All the samples were rinsed with 0.9% NaCl solution and kept stored at −70 °C. All of the above procedures were uneventful. The peripheral blood samples were used for liver parameters measurements using standard biochemical procedures. Endotoxin measurements with ELISA were performed using the peripheral, aorta and portal blood samples.

The brain samples were used for the study of the oxidative stress.

### Oxidative stress (OS) assessment

#### Sample treatment for OS evaluation

Brainstem, cerebellum and hemisphere samples from each animal are excised, washed in 0.9% NaCl, mixed with 3 × vol homogenization buffer in the tube of a Dounce type glass homogenizer, and homogenized. Then, the homogenate is transferred (with a Pasteur pipette) into a 2-ml-microcentrifuge tube, centrifuged at 16,000 g for 5 min at 4 °C, and the resulting supernatant (clear homogenate) is collected, and treated for isolation of total proteins following a modification of a previous published protocol ([20]—in Additional file 1), as follows:

A 600 μl homogenate is mixed with 12 μl 1% DOC (0.02% final concentration), and incubated for 15 min at RT, followed by the addition of 68 μl 100% TCA (10% final), incubation in an ice-water bath for 30 min, and centrifugation for 5 min at 16,000 g and 4 °C. The supernatant is discarded, and the resulting protein pellet is washed. The protein pellet is treated for removing any remnants of DOC and TCA by 3x-washes with 500 µl −20 °C-cold acetone (by dispersing the pellet with the curved end of a narrow glass rod), followed each time by centrifugation for 5 min at 16,000 g and 4 °C (and acetone discarding). Any acetone remnants in the protein pellet are evaporated by the SpeedVac for 5 min, and the powder-like total protein pellet is processed (it can be also stored at −40 °C, until further treatment). The total protein pellet is solubilized in a minimum volume (~ 50 µl) 50 mM NaOH and used for the determination of peroxidized lipid- and free radical-induced protein oxidative modification (see below), after using a 10 × dilution for protein quantification by a sensitive protein method described elsewhere [21].

### OS evaluation

OS is evaluated by the following OS-induced protein oxidative modification specific assays:

#### Peroxidized lipid-induced protein oxidative modification

Peroxidized lipid-induced generation of protein-bound (Pr) malondialdehyde (MDA), designated PrMDA, is measured by the MDA-specific thiobarbituric acid-based photometric assay, described elsewhere [22]. MDA is the very reactive aldehydic end product of lipid peroxidation, which oxidatively modifies total proteins by binding to them.

#### Free radical-induced protein oxidative modification

Protein carbonyls (PrC = O) are measured by the specific rhodamine B hydrazide (RBH)-based fluorometric assay, described elsewhere [14]. PrC = O result from the free radical-generated carbonyl groups (–C = O) on them.

### Statistical analysis

Descriptive statistics were reported either as mean ± standard deviation (SD) or median (min–max). Normality of data distribution was assessed with the Shapiro–Wilk test. Comparisons among groups were performed using analysis of variance (ANOVA) or the Kruskal–Wallis test, as appropriate, with the Bonferroni correction. Results were reported as mean ± SD when ANOVA was used and median (min–max) when the Kruskal–Wallis test was used. All tests were two-tailed. Results were considered statistically significant if p-value was less than 0.05.

## Supplementary Information

**Additional file 1**. Equipment, Reagents and Solutions used for Oxidative Stress Assessment.

## Abbreviations

ALP: Alkaline phosphatase
BBB: Blood brain barrier
CBD: Common bile duct
CRP: C-reactive protein
GGT/γGT: Gamma-glutamyl transferase
LDH: Lactate dehydrogenase
OS: Oxidative stress
PrC = O: Protein carbonyls
PrMDA: Protein-bound malondialdehyde
RBH: Rhodamine B hydrazide
RNS: Reactive nitrogen species
ROS: Reactive oxygen species
SGOT: Serum glutamic oxaloacetic transaminase
SGPT: Serum glutamate pyruvate transaminase
