# Supplementary material for: Time progression and regional expression of brain oxidative stress induced by obstructive jaundice in rats
Source: Lab Anim Res. 2022 Nov 24;38:35. doi: 10.1186/s42826-022-00146-z (PMC9701014; doi:10.1186/s42826-022-00146-z)
Supplement: Supplementary file 1 — Additional file 1. Equipment, Reagents and Solutions used for Oxidative Stress Assessment. [file 42826_2022_146_MOESM1_ESM.docx]

**Supplement – Equipment, Reagents and Solutions used for Oxidative Stress Assessment**

**Equipment**

- Balance (Kern, model 770/65/6J)
- Double-beam spectrophotometer (Shimadzu, model UV-1800)
- Magnetic stirrer (FALC, model F30)
- Microcentrifuge (Thermo Scientific, model Pico 17)
- Microcentrifuge clear tubes, 1.5 and 2 ml (VWR, cat. no. 89000-028)
- Micropipettes 2.5 to 10 μl, 20 to 200 μl, and 0.1 to 1 ml, adjustable volume (Eppendorf Research)
- Microcuvette for absorbance measurements, 12.5x12.5x45 mm external dimensions, 4 mm internal window width, and 9 mm bottom thickness, 1.16 ml, quartz; Starna 9/B/9/Q/10)
- Microcuvette for fluorescence measurements (45x4 mm, 0.5 ml, quartz; Starna SOG/Q), fitted in a Starna, FCA 4 adapter
- Spectrofluorometer (Shimadzu, model RF-1501)
- SpeedVac™ Vacuum Concentrator (CHRIST, model RVC 2-18), connected to a vacuum pump (KNF, N 820.3 FT.18)
- pH meter (Metrohm, model 827 pHlab)
- Refrigerated microcentrifuge (Hermle, model Z233 MK-2)
- Sonicator (equipped with a 2-mm-diameter MS2 microtip, Dr Hielscher GmbH, model UP-50H, set at 60% power intensity)
- Vortex (FALC, model MIX 10)

**Reagents**

- Acetone (≥ 99.8%; Sigma-Aldrich, cat. no. 34850) **caution**, *highly flammable and harmful*
- Butylated hydroxyanisole (BHA; Sigma, cat. no. B1253)
- Deoxycholic acid, sodium salt (DOC; Sigma, cat. no. D6750)
- Ethanol absolute (EtOH; Merck, cat. no. 1.00983) **caution**, *highly flammable*
- Ethylenediaminetetraacetic acid, disodium salt dihydrate (EDTA; Merck, cat. no. 108418) **caution**, *toxic*
- Hydrochloric acid (HCl, 37% w/w; ChemLab, cat. no. CL00.0310) **caution**, *corrosive*
- Sodium hydroxide pellets (NaOH; Merck, cat. no. 106498) **caution*,*** *toxic, environmental hazard*
- Sodium phosphate dibasic dihydrate (Na_2_HPO_4_•2H_2_O, ≥ 99.0%; Sigma cat. no. 71643)
- Trichloroacetic acid (TCA; Merck, cat. no. 100807) **caution**, *corrosive*
- Water, ddH_2_O, purified by a Milli-Q system (Millipore Corp)

**Standard solutions**

- **0.9% NaCl**: Prepare 100 ml by dissolving 0.9 g NaCl in 99.5 ml ddH_2_O.
- **Homogenization buffer, pH 7.2** (sodium phosphate 10 mM, 1 mM EDTA, 1 mM BHA, 0.15% v/v EtOH**)**: Prepare 200 ml by dissolving 0.356 g Na_2_HPO_4_, 0.074 g EDTA in 195 ml ddH_2_O. In 360 μl absolute EtOH, dissolve 0.043 g BHA by vigorous vortex. Add dropwise 300 μl of the BHA solution into the 195 ml buffer while stirring with a magnetic stirrer. Adjust pH at 7.2 by addition of 10 M HCl and add ddH_2_O for 200 ml final volume.
- **1% DOC**: Prepare 1.5 ml by dissolving 1.5 mg DOC in 1.5 ml ddH_2_O
- **100% TCA**: Prepare 10 ml by dissolving 10 g TCA in 3.8 ml ddH_2_O. Long storage at 4°C.
- **Cold acetone**: Acetone (~50 ml) is cooled down to -20°C.

**50 mM NaOH**: Prepare 20 ml by dissolving 0.04 g NaOH in final 20 ml ddH_2_O.
